# A responsible AI framework for infection surveillance in low-resource settings: ethics, opportunities and threats for LMICs (EOT-LMICs)

**DOI:** 10.1093/inthealth/ihag033

**Published:** 2026-04-06

**Authors:** Mamdooh Alzyood

**Affiliations:** School of Psychology, Social Work and Public Health, Faculty of Health, Science and Technology, Oxford Brookes University, Headington Campus, Oxford OX3 0BP, UK

**Keywords:** artificial intelligence, global health, healthcare-associated infections, implementation science, infection surveillance, low- and middle-income countries

## Abstract

The integration of artificial intelligence (AI) into infection surveillance represents a significant shift in public health; however, most AI frameworks and implementation guidance neglect the distinct constraints of low- and middle-income countries (LMICs). This commentary presents the ethics, opportunities, and threats for LMICs (EOT-LMICs) framework, developed through synthesis of the literature on AI adoption, digital health implementation and infection surveillance in resource-limited settings. The framework provides an integrated analytical structure: ethical foundations (equity, data governance, contextual validity) establish preconditions for adoption, opportunities (workforce augmentation, standardised surveillance, outbreak detection) define potential benefits warranting investment and threats (misclassification, infrastructure fragility, model drift, regulatory gaps) identify risks requiring mitigation before and during implementation. Aligned with the 4Ps model (precision, partnership, practice, people), this framework offers policymakers and infection prevention practitioners a pathway for context-appropriate AI implementation that prioritises local needs and human-centred care.

Health systems worldwide are transforming the detection, monitoring and prevention of infections. Artificial intelligence (AI), particularly large language models (LLMs) and natural language processing (NLP), has rapidly transitioned from theory to practice.^1^ These technologies can automate clinical data extraction from unstructured records, identify patterns that are invisible to human reviewers and provide early warning of outbreaks.^2^ Recent reviews have highlighted both the promise and challenges of AI-enhanced surveillance in African settings, including applications in antimicrobial resistance biosurveillance.^3^ These low- and middle-income country (LMIC)-relevant efforts provide important foundations; however, guidance remains fragmented across ethics, implementation and infection surveillance practice, motivating the need for a synthesising framework such as ethics, opportunities, and threats in LMICs (EOT-LMICs). Such capabilities hold transformative potential for systems facing workforce shortages and fragmented data infrastructure.

However, AI is not a universal solution. More than half of medical AI datasets originate from the USA and China, with high-income countries contributing the vast majority of training data.^4^ Health technologies developed in well-resourced contexts frequently fail when transplanted elsewhere without adaptation.^5,6^ Existing frameworks offer partial guidance but have LMIC limitations: the World Health Organization AI ethics guidelines emphasise inclusiveness but lack operational specificity for resource-constrained settings,^7^ while the non-adoption, abandonment, scale-up, spread and sustainability (NASSS) framework addresses adoption complexity but derives primarily from high-income case studies.^8^ Implementation frameworks such as the NASSS framework and the society-led AI lifecycle framework (SALIENT) guide adoption and implementation processes but do not provide an ethics-first, LMIC-specific decision gate for infection surveillance.^9^

The EOT-LMICs framework emerged from synthesis of the literature on AI adoption, digital health implementation and infection surveillance in resource-limited settings, informed by structured discussions with five infection prevention and implementation science experts from LMIC and high-income country institutions to ground the conceptual structure in frontline surveillance realities and identify context-specific barriers to AI adoption. It functions not as an abstract checklist but as an integrated analytical structure linking ethical preconditions, potential benefits and implementation risks to support decisions about whether, and under what conditions, AI adoption should proceed. Although EOT-LMICs may appear superficially similar to Strengths, Weaknesses, Opportunities and Threats (SWOT), the distinction is important: SWOT is descriptive, whereas EOT-LMICs is an ethics-first decision-support framework specifying preconditions, context-dependent opportunity claims and threats requiring mitigation before operationalisation. The 4Ps model (precision, partnership, practice, people) then provides the operational pathway for implementation.

Ethical considerations must be the foundation of AI initiatives in infection surveillance. The EOT-LMICs framework focuses on three domains: equity, data governance and validity. Equity concerns pervade every stage of AI deployment because digital access varies widely within countries and AI expertise is concentrated in high-income countries.^4^ Models trained predominantly on high-income country data embed assumptions that may not generalise elsewhere, producing predictable bias when applied to different populations. Data governance adds complexity because infection surveillance involves sensitive information; however, many LMICs lack strong regulations for data protection and cross-border data sharing. Contextual validity is equally critical; AI systems that perform well in validation studies may fail in different settings because of variations in clinical documentation, laboratory capacity, disease prevalence and care-seeking behaviour. Assessing contextual validity requires evaluating whether local data patterns, clinical workflows and population characteristics align with those used in model development. Guiding questions include whether data governance and consent expectations are sufficient for the intended use, whether contextual validity has been assessed against local workflows and epidemiology and whether equity risks (including digital exclusion and biased outputs) have been explicitly identified and mitigated.

Having established these ethical preconditions, the framework next examines the specific opportunities that AI may offer in LMIC infection surveillance contexts.

AI offers substantial potential for infection surveillance in LMICs, particularly in resource-limited areas. Many LMIC hospitals lack specialised infection prevention and control staff, making robust manual surveillance systems challenging to sustain.^10^ AI can assist human experts by screening clinical narratives, flagging potential infections and summarising findings, easing labour-intensive data extraction while preserving professional judgement. LMIC surveillance systems often use inconsistent data collection methods, hindering comparisons across facilities and time periods.^11^ AI systems that apply uniform classification criteria may enhance data comparability, supporting better burden estimation and intervention evaluation. Additionally, outbreak detection in resource-poor settings is often delayed;^12^ AI-enabled monitoring of clinical data may identify unusual patterns faster than traditional methods, potentially enhancing syndromic surveillance, especially in settings where laboratory confirmation is slow or absent. For example, an AI system screening admission notes for signs of healthcare-associated infection in a district hospital with limited microbiology capacity could flag cases for targeted investigation, reducing reliance on laboratory confirmation that may be delayed or unavailable. Guiding questions include which surveillance bottleneck is being addressed, such as case-finding, classification or reporting delays; what measurable benefit is expected and what human oversight and escalation pathway will remain in place.

Despite these opportunities, AI integration in LMIC infection surveillance entails considerable risk. Misclassification errors can divert limited resources, provoke unnecessary alarms or fail to detect outbreaks, thereby eroding trust. Reliance on fragile infrastructure increases vulnerability to system failures. The evolving nature of data and disease patterns introduces model drift, which can degrade accuracy over time, particularly in areas where retraining capabilities are scarce.^13^ The absence of robust regulatory frameworks in many LMICs increases dependence on vendors and reduces the transparency of the process. This dependence may also create vendor lock-in and sustainability risks, particularly when life cycle costs (connectivity, maintenance, upgrades and workforce training) are underestimated. Digital colonialism risks may emerge when external actors control infrastructure, governance or data value chains, limiting local ownership and reinforcing power asymmetries. To mitigate these risks, human oversight must remain central, supported by routine performance monitoring, transparent reporting of system limitations and clear governance structures that define accountability when AI outputs inform clinical or public health decisions. Guiding questions include the consequences of false positives and false negatives in the local context, how downtime and model drift will be monitored and managed and who holds accountability when AI outputs influence decisions.

The EOT-LMICs framework provides an analytical structure for deciding whether AI adoption should proceed, whereas the 4Ps model offers an operational pathway for how implementation should occur.^14^ EOT-LMICs precedes the 4Ps: ethical preconditions must be satisfied, opportunities must justify investment and threats must be addressable before operationalisation begins (Figure 1). Precision demands that AI tools perform accurately within specific epidemiological contexts, with external validation using local data. Partnerships require equitable collaboration between LMIC institutions and AI developers. This means emphasising co-design over technology transfer and shared governance rather than vendor dependency. Such partnerships must navigate inherent tensions, including the need for data sharing to improve model performance against privacy obligations and the urgency of outbreak response against requirements for rigorous validation. These tensions are not exceptions but predictable design realities; responsible implementation requires explicit, locally negotiated trade-offs that are transparently documented and revisited over time.

**Figure 1.**
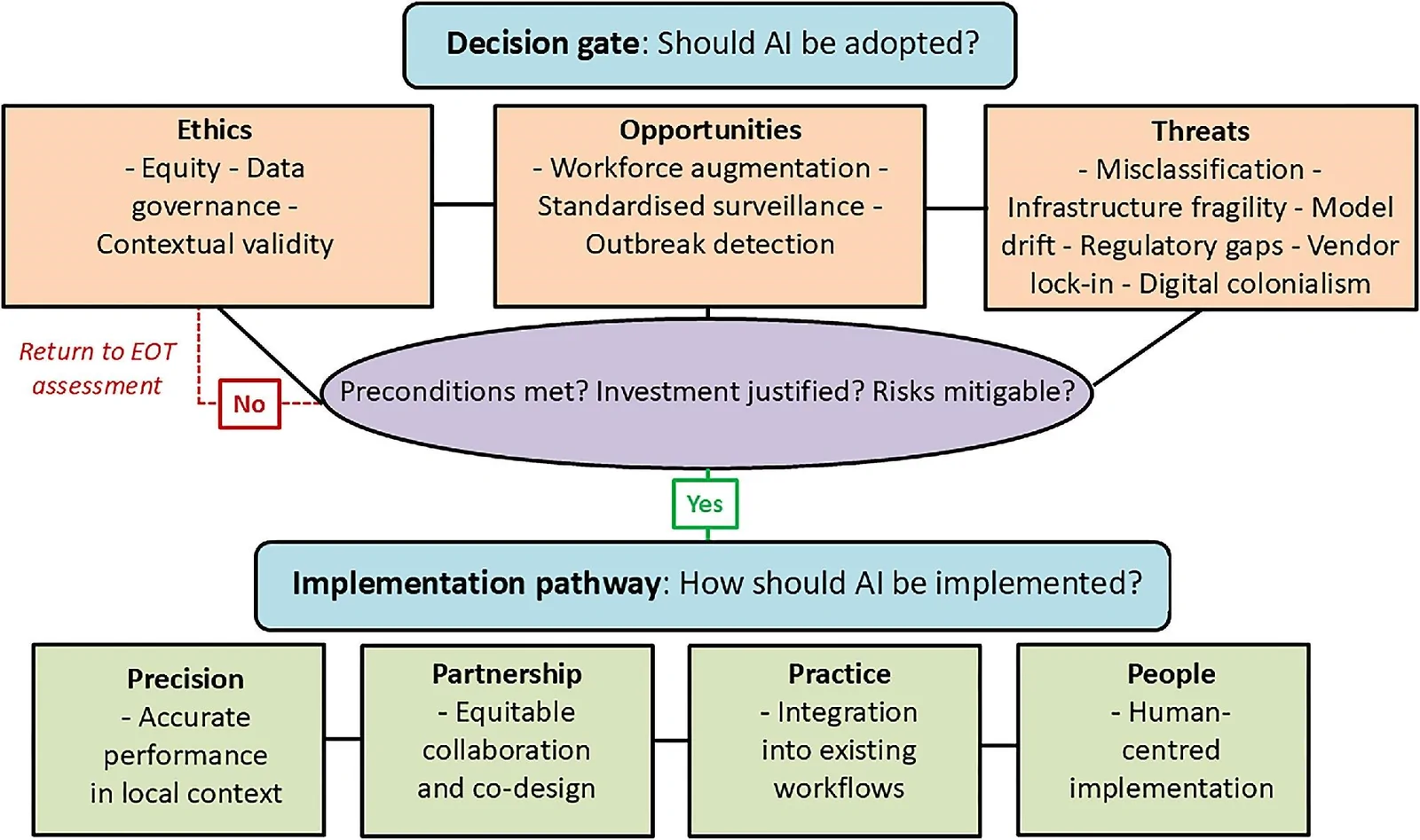
The EOT-LMICs decision framework and 4Ps implementation pathway.

The EOT-LMICs framework provides an ethics-first analytical structure for deciding whether AI adoption should proceed in LMIC infection surveillance contexts. Ethical preconditions (equity, data governance, contextual validity) must be satisfied, opportunities must justify investment and threats must be addressable before proceeding to implementation. The 4Ps model then provides the operational pathway for how implementation should occur.

Practice necessitates that AI be integrated into existing workflows rather than demanding wholesale transformation. People must remain central, as health workers and communities are not passive recipients but active stakeholders whose expertise, concerns and professional identities deserve respect and recognition. Their engagement is essential in confronting the heavy burden of healthcare-associated infections on LMIC health systems, combating antimicrobial resistance that threatens modern medicine and enabling rapid detection of emerging pathogens. AI can contribute to addressing these threats only if it is adopted with ethical integrity, operational realism and a human-centred focus. The EOT-LMICs framework provides a necessary, ethics-first analytical lens through which LMIC policymakers and infection prevention practitioners can critically appraise the suitability of AI tools for their surveillance needs before committing to implementation pathways. Its ultimate value will be determined by those working on the frontlines of infection prevention, who must judge whether this structure supports more rigorous, contextually appropriate adoption decisions. The framework is intended to be adaptable across diverse LMIC contexts, from emerging economies with developing digital infrastructure to fragile settings with minimal connectivity, with the depth of analysis calibrated to local capacity and priorities. Future work should pilot the EOT-LMICs framework in diverse LMIC health facilities, operationalise it into a practical assessment tool and evaluate its impact on AI adoption decisions and implementation outcomes. For policymakers, minimum safeguards should be required before procurement or scale-up, including clear data governance, accountability and auditability of system performance. For infection prevention practitioners, AI should be introduced with defined escalation pathways, human oversight and routine monitoring against locally relevant surveillance definitions. For researchers, priority should be given to LMIC co-designed implementation studies that evaluate not only model performance, but also equity impacts, workflow consequences and sustainability. AI must remain a tool in their hands, strengthening their work and expertise rather than displacing them.

## Data Availability

No new data were generated or analysed in support of this commentary.
